# Spatial and temporal variation of the ambient noise environment of the Sikkim Himalaya

**DOI:** 10.1038/s41598-021-04183-x

**Published:** 2022-01-07

**Authors:** Mita Uthaman, Chandrani Singh, Arun Singh, Niptika Jana, Arun Kumar Dubey, Sukanta Sarkar, Ashwani Kant Tiwari

**Affiliations:** Department of Geology and Geophysics, Indian Institute of Technology Kharagpur, West Bengal, 721302 India

**Keywords:** Seismology, Natural hazards

## Abstract

Ambient noise characteristics are perused to assess the station performance of 27 newly constructed broadband seismic stations across Sikkim Himalaya and adjoining Himalayan foreland basin, installed to study the seismogenesis and subsurface structure of the region. Power spectral densities obtained at each station, compared against the global noise limits, reveal that observed vertical component noise levels are within the defined global limits. However, the horizontal components marginally overshoot the limits due to the tilt effect. Ambient noise conditions significantly vary with different installation techniques, analysis revealing that seismic sensors buried directly in the ground have reduced long-period noise in comparison to pier installations. Tectonic settings and anthropogenic activities are also noted to cause a significant rise across short-period and microseism noise spectrum, varying spatially and temporally across the region. Day-time records higher cultural noise than night-time, while the microseism noise dominates during the monsoonal season. An assessment of the effect of the nationwide lockdown imposed due to COVID-19 pandemic revealed a significant decrease in the short-period noise levels at stations installed across the foreland basin marked with higher anthropogenic activity. Our study summarizes the overall ambient noise patterns, validating the stability and performance of the seismic stations across the Sikkim Himalayas.

## Introduction

**Figure 1:**
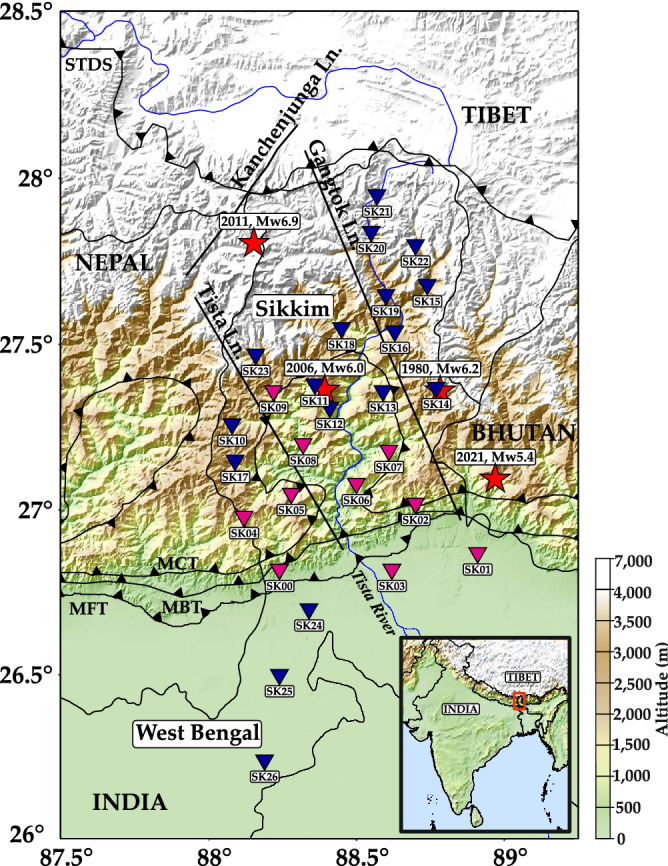
Station map of the seismic network installed in Sikkim Himalaya and regions of the Himalayan foreland basin in the northern West Bengal. Stations with the sensor installed on pier are represented with pink inverted triangles and stations with the sensor buried are represented in blue inverted triangles. Red stars represent the epicenters of major earthquakes that occurred in and around Sikkim (Source: ISC earthquake catalogue^37–39^). South Tibet Detachment System (STDS), Main Frontal Thrust (MFT), Main Boundary Thrust (MBT), Main Central Thrust (MCT), major fault lines and lineaments are also shown. Inset: Map of India highlighted with the study area. Software used to generate the figure: Generic Mapping Tools (GMT) v6.0.0^40^.

The Himalayan arc is a distinct example of an active continent-continent collision zone which is characterized by high pre-collision and post-collision rates^1^. This high impact collision led to the formation of the structurally complex Himalayan mountain range. The continued northward movement of India makes the region tectonically active. Sikkim, a state located in the northeastern part of India, lies in that part of Himalayas through which major structural features pass (Fig. 1). The recent M $\sim$ 5.4 earthquake that occurred on 5th April 2021 is a testament to the active seismicity of the region. With an aim to resolve the complex subsurface structure of Sikkim Himalaya, 27 broadband seismic stations were deployed in Sikkim and the adjoining Himalayan foreland basin (e.g., the northern part of West Bengal). Continuous monitoring and processing of the earthquake data recorded through this network will help in understanding both the genesis of earthquakes and the complex subsurface structure beneath the region^2^.

The aforementioned 27 seismic stations have been installed all across the Sikkim Himalayas and the Himalayan foreland basin, with locations chosen to provide a good azimuthal coverage (Fig. 1). Installations have been carried out in four phases (Table 1). Each seismic station is equipped with one 3-component (BHZ, BHN, BHE) broadband seismic sensor, one 24-bit digitizer, two 12V/100 Amp dry batteries to power the sensor and digitizer, two solar panels to continuously dispense charge to the batteries and two charge controllers to regulate the charge dispensed to the equipments. The stations have been continuously recording data at the rate of 50 samples per second since its installation.

**Table 1:** List of seismic stations installed, with details of the sensor type and method of installation.

Station code	Instrument type	Installation method	Installation phase	Latitude $({}^\circ )$	Longitude $({}^\circ )$	Altitude (m)
SK00	Trillium 120Q	Pier	Phase I (Apr 2019)	26.82	88.24	315
SK01	Trillium 120Q	Pier	Phase I (Apr 2019)	26.87	88.91	157
SK02	Trillium 120P	Pier	Phase I (Apr 2019)	27.02	88.70	803
SK03	Trillium 120Q	Pier	Phase I (Apr 2019)	26.82	88.62	135
SK04	Trillium 120P	Pier	Phase I (Apr 2019)	26.98	88.11	1969
SK05	Trillium 120Q	Pier	Phase I (Apr 2019)	27.05	88.28	1657
SK06	Trillium 120P	Pier	Phase I (Apr 2019)	27.08	88.50	1515
SK07	Trillium 120P	Pier	Phase I (Apr 2019)	27.18	88.61	549
SK08	Trillium 120P	Pier	Phase I (May 2019)	27.20	88.32	558
SK09	Trillium Compact	Pier	Phase I (May 2019)	27.36	88.22	1711
SK10	Trillium Horizon	Buried	Phase II (Jun 2019)	27.26	88.08	1879
SK11	Trillium Horizon	Buried	Phase II (Jun 2019)	27.38	88.36	1844
SK12	Trillium Horizon	Buried	Phase II (Jun 2019)	27.31	88.41	1356
SK13	Trillium Horizon	Buried	Phase II (Jun 2019)	27.36	88.59	1795
SK14	Trillium Horizon	Buried	Phase II (Jun 2019)	27.37	88.77	3805
SK15	Trillium Horizon	Buried	Phase II (Jun 2019)	27.68	88.74	2620
SK16	Trillium Horizon	Buried	Phase II (Jun 2019)	27.54	88.63	1441
SK17	Trillium Horizon	Buried	Phase II (Jun 2019)	27.15	88.09	2438
SK18	Trillium Horizon	Buried	Phase III (Sep 2019)	27.55	88.45	1138
SK19	Trillium Horizon	Buried	Phase III (Sep 2019)	27.66	88.60	1958
SK20	Trillium Horizon	Buried	Phase III (Sep 2019)	27.85	88.55	3585
SK21	Trillium Horizon	Buried	Phase III (Sep 2019)	27.96	88.57	4402
SK22	Trillium Horizon	Buried	Phase III (Sep 2019)	27.80	88.70	3627
SK23	Trillium Horizon	Buried	Phase IV (Oct 2019)	27.47	88.16	3928
SK24	Trillium Horizon	Buried	Phase IV (Oct 2019)	26.70	88.34	70
SK25	Trillium Horizon	Buried	Phase IV (Oct 2019)	26.50	88.24	39
SK26	Trillium Horizon	Buried	Phase IV (Oct 2019)	26.25	88.19	24

Each station records data at 50 samples per second.

The focus of this study is to analyze the quality of the data acquired from each seismic station. It is thus imperative to determine the various sources of noise that could contaminate the signal. Noise analysis will help in understanding the ambient noise conditions at the stations and the physical phenomena responsible for the same. It will also help in monitoring if there is any change in station performance or identify problems that interfere with the operation of the recording system^3^.

**Figure 2:**
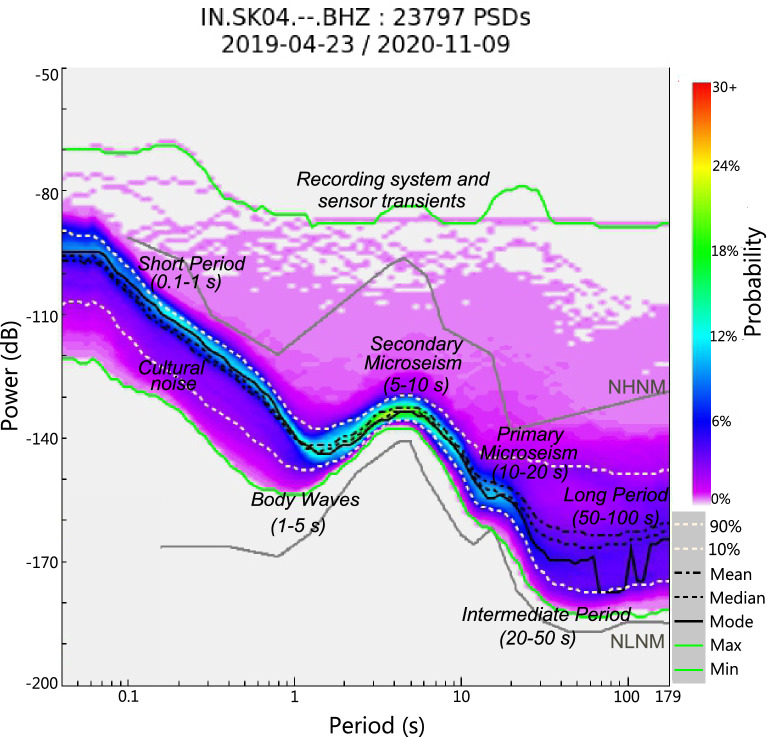
Power Spectral Density (PSD) plot of seismic background noise characteristics. The colour bar shows the probability of noise at each frequency. NHNM: New High Noise Model, NLNM: New Low Noise Model. White upper and lower dotted lines represent the 10th and 90th percentiles of the PSD, respectively. Black dashed, dotted and solid lines represent the mean, median and mode of the PSD respectively, while upper and lower solid green lines represent the maximum and minimum envelope of the PSD, respectively.

Noise spectrum is generally classified into three frequency bands: long-period (0.01–0.1 Hz), microseism (0.1–1.0 Hz) and short-period (1.0–10 Hz)^4^. However further classification of noise leads to six periods bands (Fig. 2): short-period (0.1–1 s), body wave dominant period (1–5 s), secondary and primary microseism (5–10 s and 10–20 s), intermediate period (20–50 s) and long-period (50–100 s)^5^. Atmospheric wind, volcanic activity, running water, surf, and tilt induced due to changes in wind and atmospheric pressure generate noise in the long-period band. It is observed that the horizontal components of the broadband seismometers are mostly affected by the long-period noise^6–9^. Microseism band consists of noise generated due to the activity of ocean waves along the coasts. Primary microseisms are generated due to the interaction of oceanic waves with the coast. Secondary microseisms are generated due to the superposition of ocean waves travelling in opposite directions^10^. It is thus predominant in stations installed near coastal regions. Seasonal changes also affect microseism noise. Short-period band comprises of noise generated due to human activities, automobile traffic, industrial machinery, etc.,which is broadly classified as cultural noise (0.2–0.8 s). This causes diurnal variation in the short period noise levels. River currents also contribute to noise generated in the short- period band^11^. The other sources of noise which does not get classified in the above frequency bands are oceanic storm events, quiet periods of cultural noise, system transients, data gaps, calibration pulses, sensor glitches, automatic mass recenters and earthquake signals^4^.

To determine the various sources of noise in Sikkim Himalaya and its influence on the recorded data, we have analyzed the noise spectrum in multiple ways. We have considered factors such as different installation methods, variations in geography, population density of the region, time of the day, and time of the year and its influence on the noise levels. We have also studied the effect of the nationwide lockdown imposed during COVID-19 global pandemic on the ambient noise levels in the Sikkim Himalaya and the Himalayan foreland basin.

## Tectonic settings of Sikkim Himalaya

The Himalayas were formed as a result of a continent-continent collision of the Indian and Asian plates in the Cenozoic era. This impactful collision led to the formation of not only the Himalayan orogenic belt but also the Himalayan foreland basin^12^. During the collision, the flexure formed as a result of the loading of the Eurasian plate onto the Indian plate, gave rise to the foreland basin in the south. The initial high impact collision resulted in the formation of prominent thrust faults such as the South Tibet Detachment System (STDS), Main Himalayan Thrust (MHT), Main Central Thrust (MCT), Main Boundary Thrust (MBT), and Main Frontal Thrust (MFT). MHT is a subsurface feature which is characterized as a décollement plane along which India underplates Tibet. At depth MCT, MFT and MBT eventually merge into MHT^13^. Post the collision, the continuous northward movement and convergence of India at a reduced rate resulted in the formation of many strike-slip faults orthogonal to the compression planes of the major thrusts^14^. The foreland basin system is also active because of this convergence, due to which it is filled with unconsolidated sedimentary deposits brought down by the transverse flow of the rivers in the Himalayas^15^.

In India, all of the aforementioned features pass through the Sikkim Himalayas. To add to the structural complexity, MCT traces a sinusoidal curve in Sikkim and exposes a number of thrust sheets^16^. A number of transcurrent faults lie between the curved MCT and linear MBT^17^. Hazarika et al.^18^ have proposed that the crustal shortening has been mainly assisted by the transverse tectonics in Sikkim Himalaya in lieu of the recent underthrusting. Presence of such striking structural features has given rise to a different seismicity pattern in Sikkim. Rest of the Himalayas have deeper origin earthquakes with shallow angle thrust fault mechanism, which is associated with the underplating of Indian plate beneath Tibet along the MHT. But the earthquakes originating in Sikkim Himalaya have both deep and shallow origins. Deep earthquakes originate in the north of Sikkim along the dipping plane of MHT. Shallow origin earthquakes having strike-slip fault mechanism are associated with the multiple transcurrent faults between MCT and MBT^16,19–22^.

As MCT, MBT and MFT eventually merge into the single northward dipping MHT, slips generated during the shallow earthquakes on the Himalayan front contribute to the underthrusting along the MHT^23^. The study of these earthquakes is thus crucial to delineate the possible geometry of MHT and to locate the locked portions of the fault and areas where stress and strain accumulate during the interseismic period.

## Method

Power Spectral Density (PSD) is the most commonly used tool for quantifying seismic noise. It determines the distribution of the power with frequency. McNamara et al.^24^ developed an approach which uses Probability Distribution Function (PDF) to display the PSD. PASCAL Quick Look eXtended (PQLX) is an open-source software package developed by USGS using this method^25^. Using waveform data and response files as input, PQLX Server computes the trace statistics, PSD and PDF, and stores it into a MySQL database. The results stored in the database can then be accessed by the client side GUI to display the noise spectra along with the global noise limits. For this study, we have used data recorded between April 2019 and November 2020 at the 27 broadband seismic stations in Sikkim Himalaya and its adjoining Himalayan foreland basin in northern West Bengal. Raw data (mini-SEED format) was used without removing any earthquake signals, glitches, spikes or system transients.

Data pre-processing involves deconvolving instrument transfer function from the time segments to obtain the ground acceleration that can be directly compared with the New Low Noise Model (NLNM) and New High Noise Model (NHNM)^26^. The hour long data segments are overlapped. Mean and long-period trends are removed to reduce the number of operations and long-period contamination. The segments are then tapered using a 10% sine function. This smoothens and minimizes the discontinuities present at the start and end of the time series data. The segments are further transformed using Fast Fourier Transform (FFT) to obtain the amplitude spectrum. The amplitude spectrum is squared to obtain the power spectrum. PSDs are gathered by binning periods and powers. These power-period bins are then normalized to obtain the PDF. 10th and 90th percentiles of the PSD distribution are defined as the station noise baseline envelope^3^.

PSDs for all three components at each station have been computed. Probability statistics like mean, mode and median are also plotted to interpret the plots better. PSDs for the two different installation methods are compared. Variations in ambient noise levels due to geography of the region are studied. PSD plots of day-night variations are generated to determine the effect of cultural noise. The effects of the seasonal variations are also studied. Further, the effect of the nationwide lockdown due to the COVID- 19 global pandemic on the ambient noise levels has also been analyzed.

## Results and discussion

**Figure 3:**
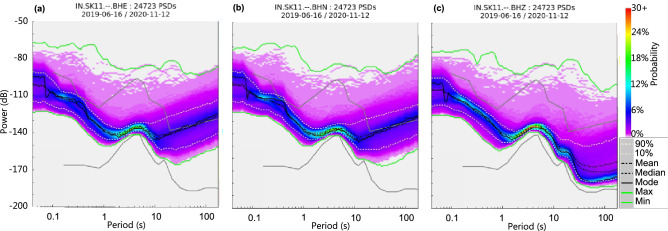
Power Spectral Density (PSD) of (**a**) E-component, (**b**) N-component and (**c**) Z-component at station SK11 for the data collected from June 2019 to November 2020. Long-period noise levels are higher in horizontal components (E, N) as compared to the vertical (Z) component.

PSDs have been computed for data recorded at all the 27 seismic stations. Noise levels recorded at all the stations fall within the NLNM and NHNM limits defined by Peterson^26^. 10th and 90th percentile lines also lie within the NLNM and NHNM limits. Statistical quantities like mean and median mimics the 90th percentile in all the cases, while mode is seen to mimic the 10th percentile line. However, some anomalous deviations have been observed in some stations, e.g., SK04 (Fig. 2) which can be accounted for by the sensor transients^4^. For analysing the overall noise spectrum, we have used the PSDs generated for vertical components of the seismometer since different types of noise can be characterized better in data recorded on the vertical component. Horizontal components tend to be noisier^27^, especially in the long-period band (Fig. 3), since it is sensitive to tilt induced noises caused by thermal variations, barometric effects or strong winds.

### Comparison of sensor installation methods

The 27 broadband seismic stations in our study area are equipped with 4 different types of sensors as indicated in Table 1. The Trillium 120P, Trillium 120Q and Trillium Compact sensors are installed above the ground, while Trillium Horizon sensors are installed below the ground in a dug out hole. The sensors installed above the ground are placed on a rectangular concrete pier inside a concrete housing with an air gap surrounding the pier. The air gap was maintained between the edge of the pier and the concrete walls to avoid the induction of the ambient noise vibrations on the seismometers. Therefore, preventing transfer of the effect of wind acting on the walls of concrete housing to the pier, reducing the induced tilts. The second format of installations placed the Trillium Horizon sensors 3 feet below the ground in a dug out hole. The sensors have been placed on a hard-rock and the resulting gap duly filled with sand to conceal the sensor completely, thus creating a buffer. This buffer shields the sensor from wind and surface effects. To study the variation in noise levels due to different installation methods, we have considered the median PSDs. Median PSDs are specifically used since they are the central estimates of the background noise recorded at any station, which does not get affected by the spikes due to earthquake signals^28^.

**Figure 4:**
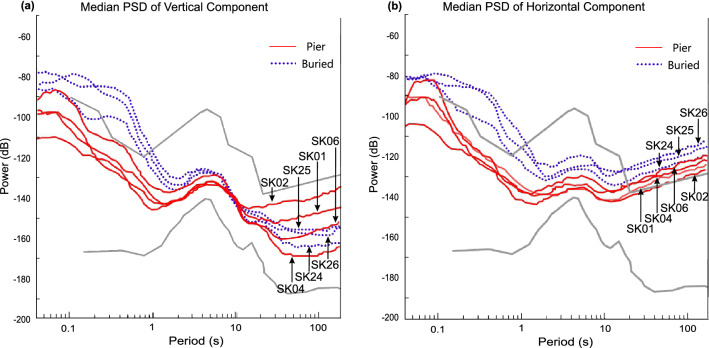
Median Power Spectral Density (PSD) plots to compare noise levels recorded at the sensors based on their installation techniques. The median PSDs of sensors recording long-period noise higher than the upper global noise limit (NHNM) in the horizontal components are plotted. Red solid lines represent median PSDs obtained using data recorded at stations where the sensor was installed above the ground on the pier. Blue dotted lines represent median PSDs obtained using data recorded at stations where the sensor was buried underground. (**a**) Median PSDs observed at the vertical component of 7 stations. (**b**) Mean of the Median PSDs observed for each horizontal component of 7 stations.

We have plotted the median PSDs of the horizontal and vertical components separately to compare the noise levels recorded in these components at all stations (Supplementary Fig. 1). Here we have shown the variations of stations at which the long-period noise in the horizontal components lie above the NHNM (Fig. 4). The median PSD plots of the vertical component show that the long-period noise levels are higher at stations where the seismic sensors have been installed on a pier above the ground than those which have been installed below the ground (Fig. 4a). The median PSD plots of horizontal components show that the noise levels in the long-period band are generally high at all the stations (Supplementary Fig. 1b), with a few (SK01, SK04, SK06, SK24) lying marginally above the global noise limit (Fig. 4b). Our observation is that the horizontal component noise levels are comparable for both types of installations. This validates that the precautions taken in order to reduce the effect of tilt induced noise in the horizontal component have been beneficial.

**Figure 5:**
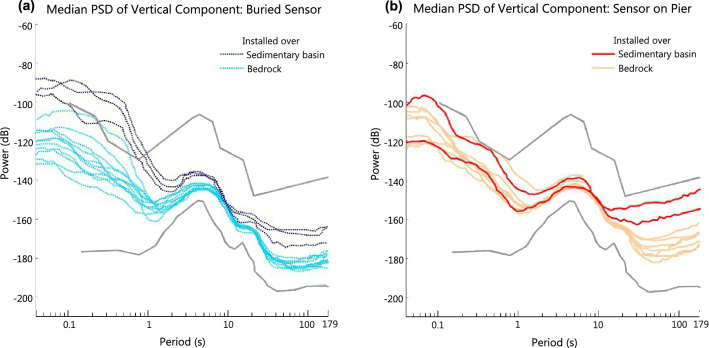
Median Power Spectral Density (PSD) plots to compare noise levels recorded at the sensors based on the surface on which they were installed. (**a**) Median PSDs observed at the vertical component of sensors buried underground. Deep and light blue dotted lines represent median PSDs obtained using data recorded at stations located over sedimentary basin and bedrock respectively. (**b**) Median PSDs observed at the vertical component of sensors installed on a pier. Red and pink solid lines represent median PSDs obtained using data recorded at stations located over sedimentary basin and bedrock respectively.

Higher than average levels of long-period noise recorded in the vertical component observed across the seismic stations in the study area is riveting. To understand the source of this high long-period noise, we consider the composition of the subsurface material at the seismic stations’ site. The subsurface material over which the station is installed affects the ambient noise levels recorded at the station. If the subsurface material is unconsolidated, the higher amplitude of ground motion induces higher long- period noise in both the horizontal and vertical components of the seismometer^29^. The stations located in Sikkim are installed over bedrocks of the Lesser Himalaya sequence (eg., SK12, SK13) and Greater Himalaya sequence (eg., SK16, SK23), as opposed to the stations located in northern part of West Bengal which are lying over the unconsolidated sediments of the Himalayan Foreland basin (Fig. 1). This explains the slightly higher noise recorded in the horizontal component at the stations (SK01, SK02, SK24) which are located over the sedimentary basin (Fig. 4b). Median PSD plot of the vertical component shows that stations installed over the sedimentary basin record higher noise levels in the long-period band than those installed over bedrock (Fig. 5). Sensors buried in unconsolidated sediments record higher noise in the long-period band ($\sim$ − 150 dB) compared to the noise levels ($\sim$ − 175 dB) recorded by sensors buried in bedrock (Fig. 5a). Likewise, sensors installed on pier above sediments record higher noise levels ($\sim$ − 135 dB) compared to the sensors installed on pier ($\sim$ − 150 dB) above bedrock (Fig. 5b). Considering the different installation techniques, we observe that sensors buried in sediment/bedrock record $\sim$ 20 dB lower long-period noise levels than sensors installed on a pier overlying sediment/bedrock. Thus, it can be safely assumed that an underground installation effectively reduces the ambient noise levels at any station.

### Geographic variation

**Figure 6:**
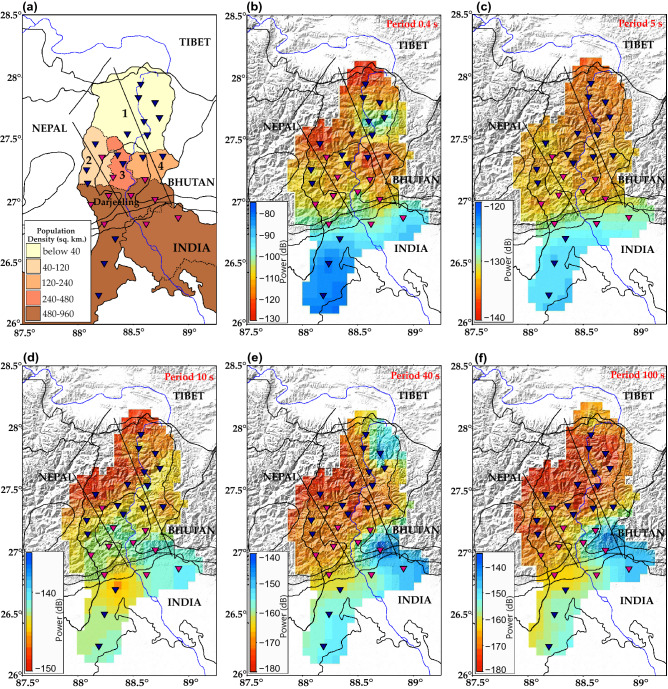
Representation of (**a**) the population density across the study area, and median Power Spectral Density (PSD) values of the ambient noise in the vertical component at different periods (**b**) 0.4 s, short-period, (**c**) 5 s, secondary microseismic period, (**d**) 10 s, primary microseismic period, (**e**) 40 s, intermediate period and (**f**) 100 s, long-period noise. The colour scales in each sub-figure, (**b**–**f**), representing PSD (dB), are different for each period to highlight the changes in the ambient noise levels across the Sikkim Himalaya. Stations with sensors installed on pier are represented with pink inverted triangles and stations with sensors buried are represented in blue inverted triangles. MFT: Main Frontal Thrust, MBT: Main Boundary Thrust, MCT: Main Central Thrust, 1: North Sikkim, 2: West Sikkim, 3: South Sikkim, 4: East Sikkim. Software used to generate the figure: Generic Mapping Tools (GMT) v6.0.0^40^.

As we move from the Himalayan foreland basin in the south to the Sikkim Himalayas in the north, the geography and population density changes gradually. The geography changes from low-lying plains in the south to high altitude mountains in the north. 2011 census data of India (Source: https://www.census2011.co.in/) reveals that the Himalayan foreland basin areas of northern West Bengal are densely populated (622 persons per sq.km). The Darjeeling Himalayas has 586 persons per sq.km, compared to the meager 117 and 10 persons per sq.km in south and north Sikkim, respectively (Fig. 6a). Overall, the population density in the southern region of our study area is much higher than that in the north. We have spatially plotted median PSD values observed at the vertical component at different periods, 0.4 s, 5 s, 10 s, 40 s and 100 s, to evaluate the variation of noise at short-period, secondary microseism, primary microseism, intermediate period and long-period, respectively^5^. The spatial plots were generated by plotting the median PSD values, obtained for each station at the aforementioned periods, at the corresponding geographical location of the station using the nearest neighbour interpolation method with a search radius of 18 km. A comparative study of the spatial median PSD plots shows that short-period and cultural noise is higher in the data recorded at stations located in the south than for those located in central and north Sikkim Himalaya (Fig. 6b). Microseism noise is higher for stations in the south which are located over the Himalayan foreland basin (Fig. 6c,d). Long-period noise is generally low at all the stations except for a few stations in south (Fig. 6e,f). To determine the said sources of noise in different regions, we consider the geographic and anthropogenic features near the 27 seismic stations installed in the study region.

Stations SK01, SK02, SK24, SK25 and SK26 are situated in the Himalayan foreland basin (Fig. 1). The sediments carried by the north-south flowing Tista River get deposited in this region. Being a plain land region, the population density is also higher (Fig. 6a). Analysis of the short-period noise across the Himalayan foreland basin (Fig. 6b) reveals that the noise levels at 0.4 s period is high ($\sim$ − 90 ± 10 dB). This can be accounted by the higher cultural noise levels owing to the higher population density. The microseism noise band (5–10 s) reveals higher levels of noise (> − 130 dB, Fig. 6c,d). This is accounted for by the sedimentary deposits that occupy this region. Higher reflection and refraction of surface waves in these sediments give rise to noise in the microseism frequency band^30^. In the intermediate and long-period band spatial maps (Fig. 6e, f), higher noise levels ($\sim$ − 155 dB) are observed. As discussed earlier, the above ground installation of the sensor at these stations makes the sensor more prone to tilts induced due to thermal and barometric variations which generates noise in the long-period range.

Stations located in the central Sikkim Himalaya, SK17, SK11, SK12, SK13, etc, are installed at higher altitudes and in remote locations (Fig. 1). In the short-period (0.4 s) (Fig. 6b), we observe that noise levels are low ($\sim$ − 115 dB) owing to the lower population density (Fig. 6a). In the microseism periods (Fig. 6c,d), lower noise ($\sim$ − 133 dB) is observed. This is because the surface material changes from sedimentary deposits to rocks as we move from south to north. In the long-period band (Fig. 6f), very low noise levels ($\sim$− 170 dB) are observed. This exceptionally low long-period noise enables the sensors here to record the Earth’s “hum” too^31^.

Stations located in the northern part of Sikkim Himalaya, SK16, SK19, SK20, SK21, SK22, etc, are located at high elevations (> 3600 m, Table 1). Given the high altitude of the region, and harsh weather conditions, the population density is very low (Fig. 6a) leading to low noise levels ($\sim$ − 120 dB) in the short-period band (Fig. 6b). However, fluctuations in the Tista river current^11^ flowing in close proximity to stations SK16 and SK19 leads to higher noise ($\sim$ − 95 dB) in the short- period band (Fig. 6b). Stations in the northern part of the study region (e.g. SK21, SK22) are prone to strong winds leading to higher noise levels to be recorded in the long-period band ($\sim$ − 145 dB) due to tilt induced in the sensors. In the microseism band (Fig. 6c), lower noise levels are observed.

Apart from the variational patterns in noise across the north-south extent of the study area, significant changes in the noise patterns have also been observed along the east-west extent. Considering the variations of noise along the Himalayan Foreland Basin, higher long-period noise levels are recorded in the east than the west. This is because the sensor at these stations are installed above the ground on a pier over the unconsolidated sediment, which as discussed in the previous section leads to higher long-period noise recorded even in the vertical component. These variations are most dominant for the long-period, while significant east-west variations are not observed for the lower periods, which are predominantly influenced by anthropogenic activities rather than geographic variations. Some variational noise patterns across the south and central Sikkim (Fig. 6e,f) are also noted where the MCT curves in the eastern section of the study area (close to station SK07, Fig. 1), towards Bhutan, notes higher noise (> − 150 dB) compared to the west (< − 165 dB), a possible effect of the seismicity of the region that is concentrated along western Bhutan^32^. Seismicity induces a tilt effect due to higher ground shaking at the instrument site^33^ leading to higher long-period noise.

### Diurnal variation

**Figure 7:**
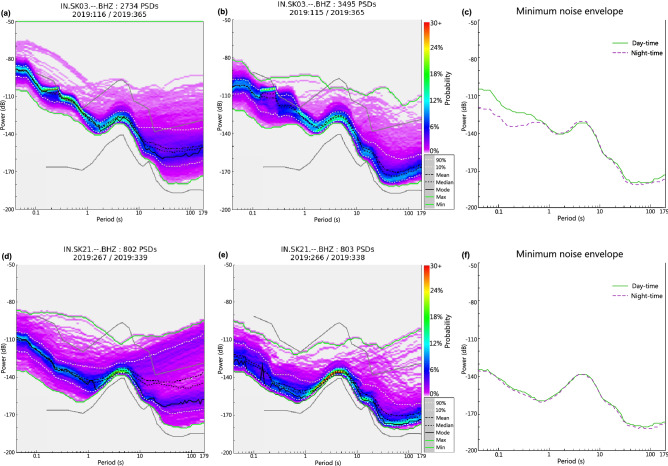
Power Spectral Density (PSD) plots for station SK03 and SK21 using (**a**), (**d**) day-time data and (**b**), (**e**) night-time data, respectively. (**c**) Minimum noise envelope obtained from the PSD plots of day-time and night-time data of SK03 shows that the cultural noise (0.2–0.8 s) is lower during night as opposed to that during day. (**f**) Minimum noise envelope obtained from the PSD plots of day-time and night-time data of SK21 shows no difference.

Variations in noise during day-time and night-time help in determining the influence of cultural noise at any seismic station. As population increases, cultural noise tends to be higher during day-time and lower during night-time. This change is often observed in the short-period band (0.2–0.8 s). For the study of day and night variation, we have considered PSDs generated on the vertical component of station SK03 and SK21 (Fig. 7). Station SK03 is located in the southern region of the study area (Fig. 1) which has a higher population density as opposed to station SK21 which is located in the northern region of the study area with a lower population density (Fig. 6a). Day- time PSDs (Fig. 7a,d) have been generated using data recorded between 05:00 a.m. to 11:00 a.m., and night-time PSDs (Fig. 7b,e) using data recorded between 11:00 p.m. and 05:00 a.m. To better illustrate the difference in noise levels, the minimum noise limits obtained from the PSD plots of day-time and night-time data are plotted along with NHNM and NLNM (Fig. 7c,f). A comparison of the day-time and night-time PSD of station SK03 (Fig. 7a,b) shows a decrease in noise during night-time. The minimum noise envelope plot (Fig. 7c) further asserts this difference with a prominent reduction of noise level by $\sim$ 15 dB during night-time. On the contrary, day-time and night-time PSD plots of station SK21 (Fig. 7d,e) shows very little difference in cultural noise. The minimum noise envelope plot (Fig. 7f) shows almost the same noise level across all period bands. Thus, variations in cultural noise highly depend upon the population density of the region. Microseism noise remains unaffected during diurnal variations.

### Seasonal variation

Seasonal variations are observed to have a considerable effect on the ambient noise levels recorded in the Sikkim Himalayas. Generally, microseism noise levels increase during monsoon due to increased water level and storms. Long-period noise levels marginally increase during summer due to higher thermal variations, while increased atmospheric wind and pressure conditions during winter also raises the long-period noise levels^4^. To study the effect of seasonal variations, we generated spatial maps using the vertical component median PSD values recorded during different seasons in three frequency bands (0.4 s, 4 s, 40 s) corresponding to the short-period, microseism and long-period bands. The time series data have been sorted seasonally into summer (March–June), monsoon (July–September) and winter (November–February) months, and separate PSDs generated have been subjected to analysis.

#### Ambient noise variations in summer

**Figure 8:**
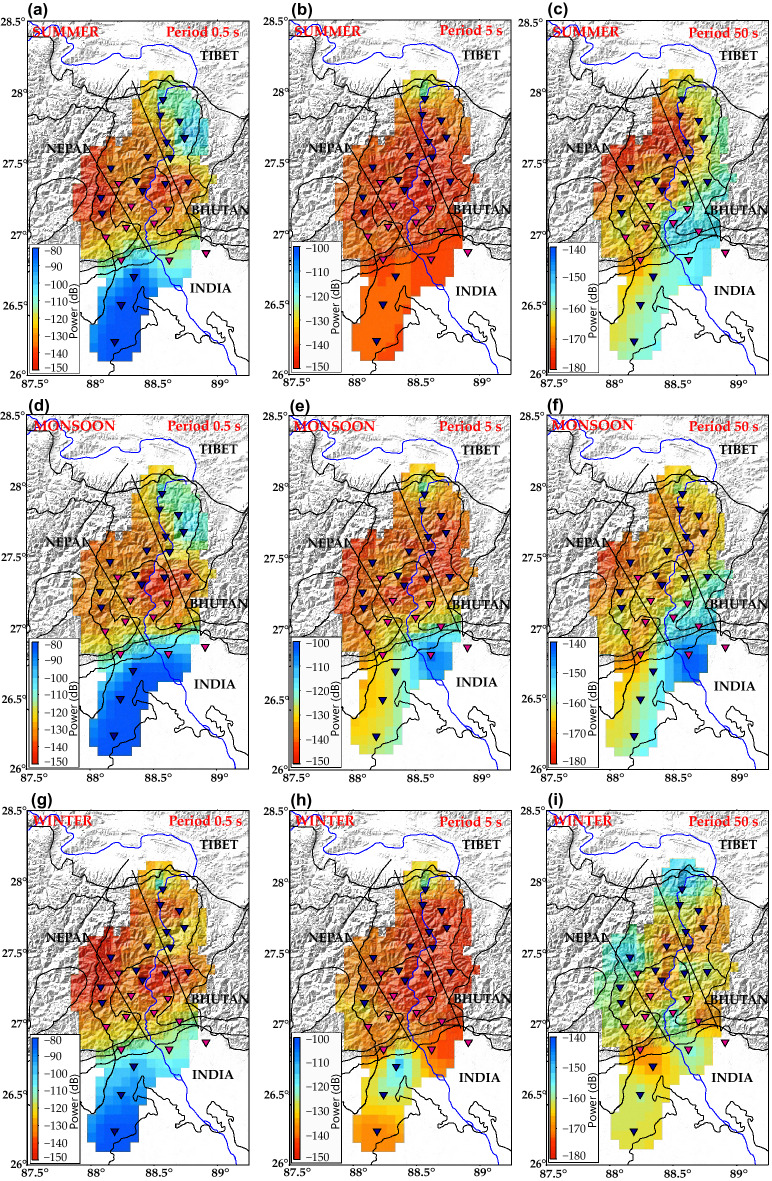
Representation of the median Power Spectral Density (PSD) values of the ambient noise recorded by the vertical component at three periods (0.4 s, 4 s and 40 s) for summer, monsoon and winter season to study seasonal variations in noise levels. (**a**), (**d**), (**g**) Noise level for summer (March-June) at three defined periods. (**b**), (**e**), (**h**) Noise level for monsoon (July-September) at three defined periods. (**c**), (**f**), (**i**) Noise level for winter (November-February) at three defined periods. The colour scales in each sub-figure, (**a**–**i**) representing PSD (dB), are different for each period to highlight the seasonal variations of the ambient noise levels across the study area. Stations with sensors installed on pier are represented with pink inverted triangles and stations with sensors buried are represented in blue inverted triangles. MFT: Main Frontal Thrust, MBT: Main Boundary Thrust, MCT: Main Central Thrust. Software used to generate the figure: Generic Mapping Tools (GMT) v6.0.0^40^.

In the short-period band, higher noise levels are observed in stations located in south ($\sim$ − 85 dB) in comparison to the north ($\sim$ − 100 dB) of the study region (Fig. 8a). Increased cultural activities during the summer months in these regions can account for this rise. Given the higher population density in the south, the cultural activity is higher. In the north, especially in the eastern region, the uncharacteristically enhanced noise levels in the short-period can be accounted for by the anthropogenic activity by the population that resides in the region, which diminishes during the winter months. Microseism noise recorded in summer is low at all the stations, varying between − 150 to − 130 dB (Fig. 8b). In the long-period band, higher noise levels are observed at the stations in the south (Fig. 8c). Thermal variation induces tilt across all the components (more pronounced in horizontal components than vertical components) causing the observed high noise levels. The stations in the southeastern region of the study area, the above ground installation over unconsolidated sediments makes them much more susceptible to tilts induced due to thermal and barometric variations causing higher long-period noise to be recorded.

#### Ambient noise variations in monsoon

In the short-period band, higher noise levels are observed at stations located in south Sikkim and northern part of West Bengal (Fig. 8d) following similar trends as the summer months. Noise generated due to cultural activities in the populated regions of the Himalayan foreland basin accounts for this observation. For stations in the northeastern part of the study area, which are located in close proximity to the Tista River, the increased turbulence accounts for the increase in short-period noise at these stations^11^. Low noise levels ($\sim$ − 145 dB) are observed in the central region. In the microseism band, higher noise levels ($\sim$ − 125 dB) are observed in the stations located in the southeastern region (Fig. 8e). Fluctuating groundwater levels in the sediments due to change in precipitation enhances the propagation of Rayleigh waves^34^, generating noise in the microseism band. For the rest of the study area, moderate to low level of microseism noise is observed. In the long-period band, higher noise levels are observed in the southeastern region (Fig. 8f). An increased storm during monsoon generating noise in the long- period range accounts for the higher noise levels observed.

#### Ambient noise variations in winter

In the short-period band, high noise levels ($\sim$ − 110 to − 90 dB) are observed in the south (Fig. 8g). Cultural noise can be accounted for by this. Compared to the noise levels observed in north during the summer and monsoon seasons, a drop of $\sim$ 20 dB in the short-period noise levels is observed in winter, owing to the inaccessibility of the region leading to lesser anthropogenic activities during the winter months. Extremely low temperatures, snow storms and reduced flow rates of the river cumulatively influence the ambient noise levels during winter. The microseism noise levels observed are lower than monsoonal months throughout the study region (Fig. 8h). Moderate microseism noise levels ($\sim$ − 130 dB) are observed in the south. In the long-period band, high noise levels (> − 150 dB) are observed in the stations located at higher altitudes in north, and western part of the central region (Fig. 8i). Increased snow storm activities in these regions generate noise in the long-period frequency range. For the rest of the study area, moderate to low ambient noise levels are observed in the long-period band.

### Variations in noise due to the global COVID-19 pandemic

Since the worldwide spread of Coronavirus disease (COVID-19) in 2020, the World Health Organization (WHO) declared it as a global pandemic^35^. Ever since then all the countries around the world have been trying various strategies to curb its spread. One of the widely used strategies is imposing a total lockdown, which restricts the transport or movement of any kind for a particular period of time. An extensive study of ambient noise levels around the world during such lockdown periods revealed that there was a significant reduction in the short-period noise levels due to the decreased anthropogenic activities^36^. In India, lockdown was announced on 21st March 2020. To determine the effect of this nationwide lockdown on the ambient noise levels recorded in the Sikkim network, spectrograms generated using data recorded at all the stations are studied. Major variations are seen for the stations SK24, SK25 and SK26, situated in the northern part of West Bengal in the Himalayan foreland basin.

**Figure 9:**
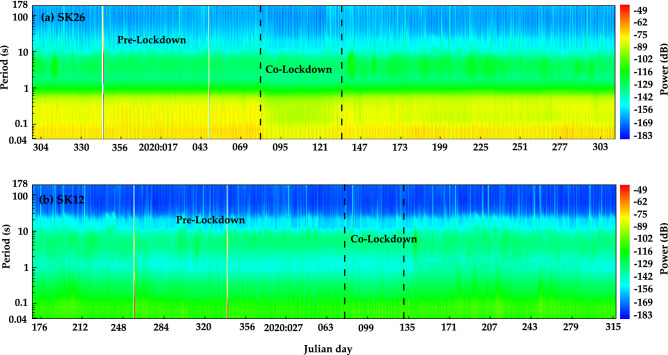
(**a**) Spectrogram plot at station SK26 to study the variations in noise due to the global COVID-19 pandemic. Spectrogram illustrates the noise level for the entire duration of data collection at the station. A decrease in short-period noise between Julian days 81 to 130 (March 22–April 22 2020). (**b**) Spectrogram plot at station SK12. No visible variation in noise is observed during the lockdown period since the station is situated in a remote location.

We analyze the noise spectrogram data recorded at station SK26 (Fig. 9a). This station is located in a residential area with many government offices, shops and markets surrounding it. The national highway and railway lines are just 1 km away. Due to higher anthropogenic activities, short-period noise levels are high at this station which is also evident from the PSD plots in Figs. 6 and 8. The spectrogram was generated using data collected from October 2019 to November 2020 at station SK26 (Fig. 9a). The nationwide lockdown, which was announced on 21st March 2020, was implemented from 22nd March 2020. From the spectrogram, it can be observed that the cultural noise drops suddenly on Julian date 81 and remains low till Julian date 130. This corresponds to the time period when complete lockdown was implemented from 22nd March–10th May 2020. After 10th May, restrictions were relaxed to allow some essential services.

Such variations are not prominent in the stations located in the central and northern Sikkim Himalayas. This is because all the stations are located in relatively remote places, for example station SK12 (Fig. 9b). Most stations are located away from the hustle of automobile traffic, which is generally less due to the lower population density ($\sim$ 10 persons per sq.km) of these places. Thus, the effect of cultural noise is generally low at all the stations in Sikkim. This corroborates to clear detection and recording of even the smallest events, increasing the data quality.

## Conclusions

This study provides a detailed characterization of the ambient noise environment of the Sikkim Himalaya and Himalayan foreland basin. Ambient noise levels at all the broadband stations are optimum and lie within the global noise limits, and the stations stabilized soon after the installation. The main concluding remarks of this study are as follows:

1. Different seismic sensors are differently influenced by the ambient noise levels, especially in the long-period band. Trillium Horizon seismometers, directly buried in the ground, have lesser impact of long-period noise compared to seismometers placed on the pier (Trillium 120P, 120Q and Compact seismometers). Higher long-period noise is observed in the horizontal components for the stations installed on the pier because of tilt induced, however, the effect is cushioned by the air gap that decouples the pier from the sensor’s housing. Higher long-period noise is observed in vertical components of sensors installed above/below unconsolidated sediments.
2. Short-period cultural noise and microseism noise levels decrease as we progress north from the Himalayan foreland basin to Sikkim Himalaya, influenced by elevation and the change in the subsurface material from loose sediments to consolidated rocks, and population density.
3. As population density in a region increases, diurnal variation in cultural noise levels become more prominent. Short-period cultural noise tends to be higher during day- time as opposed to night-time.
4. Seasonal variation is most prominent during the monsoon when an overall high microseism noise is observed. Long-period noise is higher in the north during winter because of tilt induced due to higher intensity of wind.
5. Nationwide lockdown imposed due to COVID-19 pandemic has had a major impact on the short-period noise at stations located in proximity to higher anthropogenic activity in the Himalayan foreland basin. Whereas, negligible change in cultural noise variation are observed at stations located in remote areas in Sikkim due to the scarce population density.

## Supplementary Information

Supplementary Figure 1.
