# Supplementary material for: Spatial and temporal variation of the ambient noise environment of the Sikkim Himalaya
Source: Sci Rep. 2022 Jan 7;12:274. doi: 10.1038/s41598-021-04183-x (PMC8741811; doi:10.1038/s41598-021-04183-x)
Supplement: Supplementary file 1 — Supplementary Figure 1. [file 41598_2021_4183_MOESM1_ESM.pdf]

Spatial and temporal variation of the ambient noise environment of the Sikkim Himalaya

Mita Uthaman[1], Chandrani Singh[1,*], Arun Singh[1], Niptika Jana[1], Arun Kumar Dubey[1], Sukanta Sarkar[1], and Ashwani Kant Tiwari[1]

[1]Indian Institute of Technology Kharagpur, Department of Geology and Geophysics, West Bengal, 721302, India
[*]chandrani@gg.iitkgp.ac.in

ABSTRACT

Ambient noise characteristics are perused to assess the station performance of 27 newly constructed broadband seismic stations across Sikkim Himalaya and adjoining Himalayan foreland basin, installed to study the seismogenesis and subsurface structure of the region. Power spectral densities obtained at each station, compared against the global noise limits, reveal that observed vertical component noise levels are within the defined global limits. However, the horizontal components marginally overshoot the limits due to the tilt effect. Ambient noise conditions significantly vary with different installation techniques, analysis revealing that seismic sensors buried directly in-to the ground have reduced long-period noise in comparison to pier installations. Tectonic settings and anthropogenic activities are also noted to cause significant rise across short-period and microseism noise spectrum, varying spatially and temporally across the region. Day-time records higher cultural noise than night-time, while the microseism noise dominates during the monsoonal season. An assessment of the effect of the nationwide lockdown imposed due to COVID-19 pandemic revealed a significant decrease in the short-period noise levels at stations installed across the foreland basin marked with higher anthropogenic activity. Our study summarizes the overall ambient noise patterns, validating the stability and performance of the seismic stations across the Sikkim Himalayas.

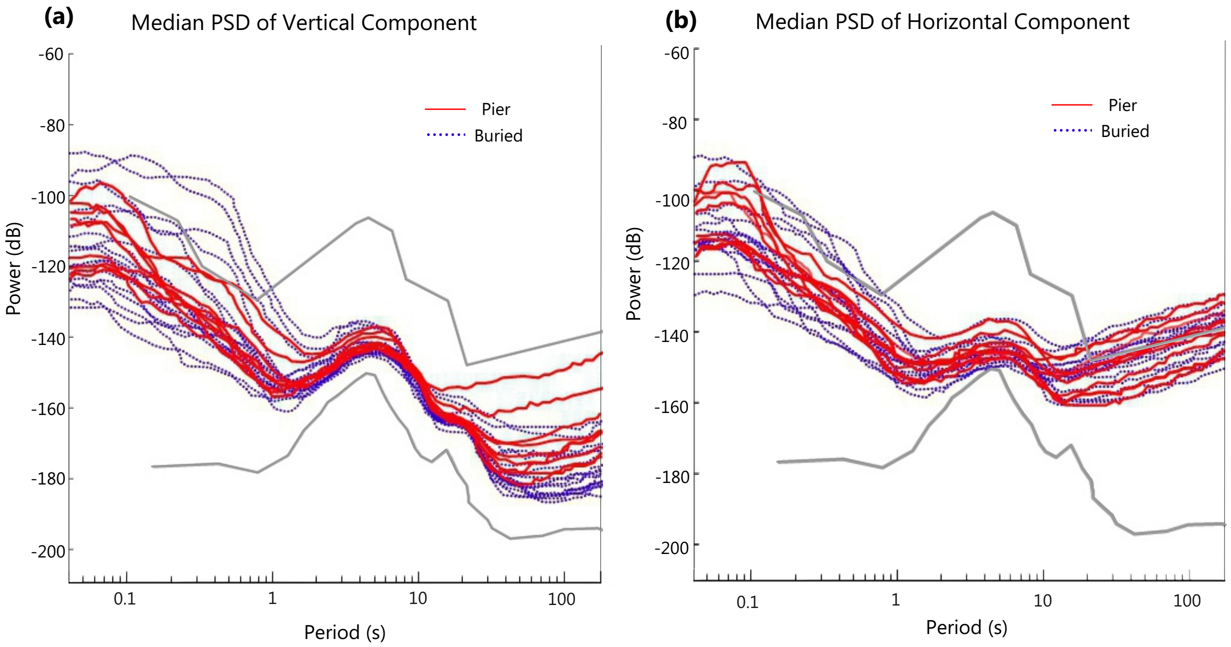

Figure 1. Median Power Spectral Density (PSD) plots to compare noise levels recorded at the sensors based on their installation techniques. Red solid lines represent median PSDs obtained using data recorded at stations where sensor was installed above the ground on the pier. Blue dotted lines represent median PSDs obtained using data recorded at stations where sensor was buried underground. (a) Median PSDs observed at the vertical component of each station. (b) Mean of the Median PSDs observed for each horizontal component of each station.
